# Supplementary material for: The Effect of BSCL2 Gene on Fat Deposition Traits in Pigs
Source: Animals (Basel). 2023 Feb 12;13(4):641. doi: 10.3390/ani13040641 (PMC9951708; doi:10.3390/ani13040641)
Supplement: Supplementary file 1 [file animals-13-00641-s001.zip › animals-2160384-supplementary.pdf]

Table S1. Primers used in study

Gene	primers	method	restriction enzyme
rs341493267	F- GCTCCTGGGAAGATGCAAC<br>R- GGAACTGGAGCACATAGGGC	DNA sequencing	
rs346079334	F- TGCGTCAGTTCAGGTCCC<br>R- TCCCCTGGACCCATAAAGC	PCR-RFLP, DNA sequencing	*AluI*
rs330154033<br>rs81333153	F-AGATCTGGCTGTGAGTGACA<br>R-ATGGATGCGGAGGTAGGC	DNA sequencing	

Table S2. The frequencies of alleles and genotypes of SNP rs341493267 and rs346079334 of *BSCL2* gene in Zlotnicka White and pigs active in Polish breeding

Year of slathering	Pig Breed	Frequencies of rs346079334 (G>T)						Frequencies of rs341493267 (A>G)					
		Genotype			Allele		HWE* (*P*-value)	Genotype			Allele		HWE* (P-value)
		GG	GT	TT	G	T		AA	AG	GG	A	G	
2017	Zlotnicka White	0.51 (37)	0.33(24)	0.16 (11)	0.68	0.32	0.047687	0.15(11)	0.38 (27)	0.47 (34)	0.65	0.35	0.162079
2021-2020	Polish Large White	0.34(58)	0.33(56)	0.33(55)	0.51	0.49	0.000012	0.05 (8)	0.41(70)	0.54 (91)	0.26	0.74	0.295348
	Polish Landrace	0.54 (108)	0.36(73)	0.10(20)	0.72	0.28	0.150601	0.05 (9)	0.43 (86)	0.53 (105)	0.27	0.73	0.096676
2020	Polish Landrace	0.55 (52)	0.32(30)	<u>0.14(13)</u>	0.71	0.29	0.032481	<u>0.08 (7)</u>	0.41 (39)	0.51 (48)	0.29	0.71	0.810452
2021	Polish Landrace	0.53 (56)	0.41(43)	<u>0.07(7)</u>	0.75	0.25	0.743323	<u>0.02 (2)</u>	0.44 (47)	0.54 (57)	0.24	0.76	0.027945
2020	Polish Large White	0.33 (22)	0.37(25)	0.30(20)	0.52	0.48	0.038318	0.07 (5)	0.45 (30)	0.48 (32)	0.30	0.70	0.571388
2021	Polish Large White	0.35 (36)	0.30(31)	0.34(35)	0.50	0.50	0.000075	0.03 (3)	0.39 (40)	0.58 (59)	0.23	0.77	0.215157

*HWE – Hardy-Weinberg Equilibrium: If p-value < 0.05 - not consistent with HWE. Not accurate if <5 individuals in any genotype group. In brackets is the number of individuals in the genotype group. In the brackets are shown the number of particular individuals

Table S3. The frequencies of alleles and genotypes of SNP rs341493267 and rs346079334 of *BSCL2* gene in Zlotnicka White and pigs active in Polish breeding

Year of slathering	Pig Breed	Frequencies of rs330154033 (A>G)						Frequencies of rs81333153 (G>C)					
		Genotype			Allele		HWE* (*P*-value)	Genotype			Allele		HWE* (P-value)
		AA	AG	GG	A	G		CC	CG	GG	C	G	
2017	Zlotnicka White	0.15 (11)	0.39(28)	0.46 (33)	0.35	0.65	0.227820	0.32(23)	0.51 (37)	0.17 (12)	0.57	0.43	0.656953
2020-2021	Polish Large White	0.05(9)	0.45(76)	0.50(84)	0.28	0.72	0.118803	0.06 (10)	0.41 (69)	0.53 (90)	0.26	0.74	0.677572
	Polish Landrace	0.06(12)	0.34(68)	0.60(120)	0.23	0.77	0.570739	0.06 (11)	0.36(72)	0.59 (117)	0.24	0.76	0.985878
2020	Polish Landrace	0.07(7)	0.36(34)	0.57(54)	0.25	0.75	0.610699	0.02 (2)	0.49 (46)	0.49 (46)	0.27	0.73	0.014043
2021	Polish Landrace	0.05(5)	0.33(35)	0.62(66)	0.19	0.81	0.896447	0.08 (9)	0.25 (26)	0.67 (71)	0.21	0.79	0.008832
2020	Polish Large White	0.09(6)	0.43(29)	0.48(32)	0.31	0.69	0.875474	0.00 (0)	0.49 (34)	0.51 (33)	0.25	0.75	0.005386
2021	Polish Large White	0.03(3)	0.46(47)	0.51(52)	0.26	0.74	0.045477	0.10 (10)	0.37 (35)	0.56 (57)	0.26	0.74	0.846003

*HWE – Hardy-Weinberg Equilibrium: If p-value < 0.05 - not consistent with HWE. Not accurate if <5 individuals in any genotype group. In brackets number of individuals in the genotype group.

Table S4. Least-square means (LSM) ± SE for important pig traits dependent on *BSCL2* genotypes for Złotnicka White

Mutacja	Traits	Genotype						GLM significance				Effect	
		LSM	SE	LSM	SE	LSM	SE	*BSCL2* P-value	X2P	Farm	sire	Additive	Dominance
		AA (n=11)		AG (n=27)		GG (n=34)						A→G	het→hom
rs341493267	Ham mass without skin and fat (kg)	7.63	0.26	7.56	0.14	7.66	0.11	0.1976	**	ns	ns	-0.03^{*}	-
	Fat thickness over back (cm)	2.96	0.22	2.87	0.11	2.90	0.10	0.1444	**	*	ns	-	$+0.12^{T}$
	Average Backfat Thickness (cm)	2.24	0.20	2.18	0.10	2.36	0.08	0.1034	**	*	ns	-	-
	Loin eye area (cm2)	42.4	0.75	43.9	1.01	43.1	1.75	0.1779	**	*	ns	-	-
	Backfat in the point K1	1.94	0.17	1.89	0.11	2.14	0.11	0.0883	***	ns	ns	-	-
	Backfat in the point C1	1.92	0.17	1.90	0.11	2.15	0.12	0.0913	**	*	ns	$+0.19^{T}$	-
	Meat percentage	52.7	1.19	52.4	0.74	52.8	0.82	0.1413	**	*	ns	-	-
	Daily gain (30-100 kg)	748	28	716	21	677	19	0.1591	-	ns	ns	$+6.53^{T}$	-
	Days in Test (days)	94	4	99	3	107	5	0.1667	-	ns	ns	-	-
rs346079334		GG (n=11)		GT (n=24)		TT (n=37)							
	Loin mass without skin and fat (kg)	4.46	0.17	4.53	0.11	4.36	0.07	0.0935	***	ns	ns	-	-
	Backfat at the lumbar I (cm)	1.97	0.21	2.13	0.12	2.31	0.09	0.0743	**	*	ns	-0.17^{T}	-
	Backfat at the lumbar II (cm)	1.83	0.19	1.92	0.12	2.00	0.09	0.1069	**	ns	ns	-	-
	Backfat at the lumbar III (cm)	2.40	0.20	2.34	0.12	2.47	0.11	0.1278	**	ns	ns	-	-
	Avarage Backfat Thickness (cm)	2.34	0.20	2.23	0.10	2.32	0.08	0.1819	***	*	ns	-	-
	Loin eye area (cm2)	43.1	1.75	44.8	0.94	42.0	0.76	0.0683	**	ns	ns	-	-
	Backfat in the point K1	1.93	0.17	1.95	0.12	2.09	0.11	0.1609	**	*	ns	-	-
	Backfat in the point C1	1.91	0.17	1.96	0.12	2.09	0.11	0.1602	**	*	ns	-	-
	Meat percentage	52.7	1.19	52.3	0.83	52.8	0.76	0.1389	***	**	ns	-	-
	Daily gain (30-100 kg)	748	28	720	22	677	19	0.1553	-	ns	Ns	$+35^{T}$	
	Days in Test (days)	94	4	99	2	106	4	0.1870	-	ns	ns	-6.26	-
rs330154033		AA (n=11)		AG (n=28)		GG (n=33)							
	Peritoneal fat (kg)	0.54	0.04	0.56	0.04	0.61	0.03	0.1776	***	*	ns	$+0.03^{*}$	-
	Ham mass without skin and fat (kg)	7.63	0.26	7.54	0.14	7.69	0.12	0.1844	**	ns	*	-	-
	Fat thickness over back (cm)	2.96	0.22	2.92	0.11	2.86	0.10	0.1519	***	*	*		$+0.11^{T}$
	Backfat at the lumbar I (cm)	1.97	0.21	2.15	0.10	2.31	0.10	0.1160	**	ns	ns	-	$+0.12^{T}$
	Backfat at the lumbar II (cm)	1.83	0.19	1.89	0.11	2.03	0.10	0.0843	**	ns	*		
	Backfat at the lumbar III (cm)	2.40	0.20	2.35	0.10	2.48	0.12	0.1877	**	ns	*		
	Loin eye area (cm2)	43.1	1.75	43.8	0.98	42.5	0.77	0.1888	***	**	ns		
	Meat percentage	52.7	1.19	52.2	0.72	52.9	0.85	0.1620	**	*	ns		

rs81333153		GG (n=12)		GC (n=37)		CC (n=23)							
	Feet mass (kg)	0.97	0.03	0.93	0.01	0.93	0.02	0.1538	***	ns	ns	+0.026^{T}	-
	Ham mass without skin and fat (kg)	7.64	0.24	7.52	0.11	7.77	0.16	0.1553	***	ns	*	-	
	Fat thickness over back (cm)	2.97	0.20	2.98	0.10	2.73	0.11	0.0938	**	*	ns	-	+0.086^{T}
	Backfat at the lumbar I (cm)	1.98	0.19	2.25	0.09	2.24	0.13	0.1437	**	ns	ns	+0.14^{T}	
	Backfat in the point C1	1.83	0.17	1.94	0.09	2.03	0.13	0.0843	**	*	ns		
	Backfat in the point K1	2.35	0.19	2.45	0.09	2.39	0.15	0.078	**	*	ns		
	Meat percentage	52.6	1.09	51.7	0.61	54.1	1.07	0.0903	**	*	ns		0.82^{T}

Mean and SE were estimated using GLM model, values with the same superscripts belong to the same statistical group (A, B = p<0.01; a, b = p<0.05), P-value in GLM significant *p< 0.05, ** p<0.01, *** p<0.001, ns – not significant, X2P - covariate for weight of the right side of the carcass, T – pvalue 0.05-0.20

Table S5. Least-square means (LSM) ± SE for important pig traits dependent on *BSCL2* genotypes for Polish Landrace

Mutacja	Traits	Genotype						GLM significance				Effect	
		LSM	SE	LSM	SE	LSM	SE	*BSCL2* P-value	X2P	Farm	sire	Additive	Dominance
		AA (n=9)		AG (n=86)		GG (n=105)						A→G	het→hom
rs341493267	Fat thickness over back (cm)	1.33	0.10	1.40	0.05	1.48	0.05	0.1078	**	ns	ns	-0.198^{*}	$+0.044^{*}$
rs346079334		GG (n=108)		GT (n=73)		TT (n=20)							
	Feet mass (kg)	1.18	0.01	1.21	0.01	1.20	0.02	0.1346	**	**	ns		
	Carcass yield (kg)	76.2	0.15	76.1	0.18	76.4	0.32	0.1147	**	**	ns	0.198^{T}	-
	Backfat in the point C1	1.45	0.05	1.50	0.07	1.71	0.19	0.1230	***	ns	ns		0.13^{*}
	Backfat in the point K1	1.50	0.05	1.51	0.07	1.75	0.17	0.1384	**	ns	ns		0.122^{*}
rs81333153		CC (n=11)		GC (n=72)		GG (n=117)							
	Carcass yield (kg)	75.9	0.35	76.0	0.20	76.4	0.13	0.1459	*	**	ns	-	-
	Peritoneal fat (kg)	0.75	0.06	0.64	0.03	0.71	0.02	0.1040	***	ns	*	-0.226^{*}	$+0.044^{T}$
	Feet mass (kg)	1.24	0.04	1.17	0.01	1.21	0.01	0.0616	***	ns	ns	-	$+0.026^{*}$
	Loin mass (kg)	8.19	0.13	8.19	0.08	8.41	0.06	0.0801	***	ns	ns	-	-
	Average backfat thickness	1.83	0.38	1.55	0.05	1.55	0.04	0.1246	***	*	ns	-0.139	-
	Backfat at the lumbar I (cm)	1.24	0.13	1.48	0.06	1.47	0.05	0.0931	**	**	ns	0.12^{T}	-
	Meat percentage	57.3	0.83	58.6	0.35	58.1	0.26	0.1907	**	**	ns	-	-0.444^{T}
	Daily feed intake (kg)	2.91	0.09	2.91	0.03	2.93	0.03	0.1665	-	ns	ns		
	Days in test (days)	106	4	102	2	106	1	0.1577	-	ns	ns		$+2.08^{T}$
	Ham pH45	6.15	0.09	6.15	0.02	6.22	0.03	0.1690	-	ns	ns		
rs330154033		AA (n=12)		AG (n=68)		GG (n=120)							
	Peritoneal fat (kg)	0.61	0.04	0.68	0.03	0.69	0.02	0.1978	**	ns	ns	-	-
	Ham mass without skin and fat (kg)	10.62	0.17	10.40	0.07	10.5	0.06	0.1023	***	ns	*	-	0.082^{T}
	Backfat at the lumbar I (cm)	1.48	0.11	1.52	0.06	1.43	0.05	0.1360	**	ns	*	-	-
	Primary cuts (kg)	27.1	0.31	26.7	0.17	26.9	0.13	0.0919	**	ns	*	-	0.173^{T}
	Fat thickness over shoulder (cm)	1.38	0.08	1.43	0.05	1.43	0.05	0.1347	**			-0.16^{*}	0.08^{T}

Mean and SE were estimated using GLM model, values with the same superscripts belong to the same statistical group (A, B = p<0.01; a, b = p<0.05), P-value in GLM significant *p< 0.05, ** p<0.01, *** p<0.001, ns – not significant, X2P - covariate for weight of the right side of the carcass

Table S6. Least-square means (LSM) ± SE for important pig traits dependent on *BSCL2* genotypes for Polish Large White.

Mutacja	Traits	Genotype						GLM significance				Effect	
		LSM	SE	LSM	SE	LSM	SE	*BSCL2* P-value	X2P	Farm	sire	Additive	Dominance
		AA (n=8)		AG (n=70)		GG (n=91)						A→G	het→hom
rs341493267	Peritoneal fat (kg)	0.45	0.05	0.59	0.02	0.64	0.02	0.1861	**	ns	ns	$+0.096^{**}$	-
	Backfat at the lumbar III (cm)	1.69	0.16	1.55	0.05	1.59	0.04	0.1622	**	*	ns	-	$+0.07^{T}$
	Avarage backfat thickness	1.84	0.12	1.68	0.04	1.72	0.03	0.1932	***	*	ns	-	$+0.05^{T}$
	Loin eye are (cm2)	55.0	2.21	60.0	0.67	59.0	0.66	0.0775	***	*	ns	$+1.98^{T}$	-1.63^{*}
	Backfat in the point C1	1.58	0.11	1.49	0.05	1.62	0.03	0.0895	**	ns	ns	-	-
	Backfat in the point K1	1.58	0.11	1.49	0.05	1.62	0.04	0.1568	**	ns	ns	-	-
	Water exudation	36.9	2.30	37.2	0.80	39.6	0.80	0.0838	-	ns	ns	-	
	Ham pH45	6.04	0.07	6.18	0.02	6.11	0.02	0.0896	-	ns	ns	-	-0.053^{T}
	Loin pH45	6.14	0.09	6.25	0.03	6.16	0.03	0.0796	-	ns	ns	-	-0.048^{T}
rs346079334		GG (n=58)		GT (n=56)		TT (n=55)							
	Carcass yield (kg)	75.7	0.17	75.9	0.15	75.6	0.16	0.1727	**	*	ns	-	-0.129^{T}
	Loin mass with skin (kg)	8.05	0.08	8.19	0.08	8.15	0.08	0.0644	***	*	ns	-	-
	Knuckle fat with skin (kg)	1.34	0.03	1.39	0.02	1.39	0.01	0.1090	***	**	ns	-0.02^{T}	-
	Fat thickness over shoulder (cm)	1.53	0.06	1.53	0.05	1.59	0.05	0.1596	***	ns	*	-	-
	Backfat at the lumbar I (cm)	1.53	0.06	1.60	0.05	1.60	0.05	0.1534	***	*	ns	-	-
	Backfat at the lumbar II (cm)	1.12	0.05	1.16	0.04	1.13	0.04	0.1960	***	*	ns	-	-
	Backfat at the lumbar III (cm)	1.59	0.06	1.60	0.06	1.56	0.05	0.1237	**	**	ns	-	-
	Backfat in the point C1	1.57	0.05	1.59	0.05	1.54	0.05	0.1162	***	ns	ns	-	-
	Backfat in the point K1	1.56	0.05	1.58	0.05	1.54	0.06	0.0660	**	ns	ns	-	-
	Meat percaentage	57.3	0.32	57.1	0.47	57.2	0.43	0.1759	***	ns	ns	-	-
	Slaughter age (days)	192	3	189	3	187	4	0.1867	-	ns	ns	-	-
	Meat color (MA)	16.2	0.14	15.9	0.11	15.9	0.13	0.1537	-	ns	ns	-0.148^{T}	-
	Ham pH45	6.09	0.03	6.18	0.03	6.14	0.03	0.0786	-	ns	ns	-	-0.06^{**}
rs330154033		AA (n=9)		AG (n=76)		GG (n=84)						-	-
	Knuckle fat with skin (kg)	1.27	0.04	1.38	0.02	1.38	0.02	0.1456	**	ns	ns	0.052^{T}	-0.26^{*}

	Loin mass without skin and fat	7.55	0.13	8.13	0.07	8.19	0.07	0.1720	**	ns	*	0.188^{T}	-0.029^{T}
	Loin eye area (cm2)	51.8	2.67	60.0	0.64	59.6	0.64	0.0703	**	*	ns	3.93^{***}	-2.06^{**}
	Backfat in the point K1	1.55	0.18	1.56	0.05	1.57	0.04	0.1143	***	**	ns	-	-
	Meat percentage	59.5	1.40	57.1	0.41	57.1	0.27	0.1076	**	ns	ns	-1.159^{*}	0.618^{T}
	Meat color (MA) redness	15.4	0.34	15.9	0.10	16.2	0.10	0.0789	-	ns	ns	0.378^{*}	-
	Meat color (MB) yellowness	2.93	0.27	2.76	0.13	3.08	0.11	0.1460	-	ns	ns	-	-
rs81333153		CC (n=10)		GC (n=69)		GG (n=90)							
	Daily gain (30-100 kg)	828	16	876	13	898	12	0.1794	-	ns	ns	35.4^{T}	-
	Daily gain (0-100kg)	612	12	637	8	653	7	0.1857	-	ns	ns	20.9^{T}	-
	Slaughter age (days)	200	4	192	3	187	2	0.1862	-	ns	ns	11.7^{**}	-4.50^{T}
	Days in test (days)	112	2	105	2	103	2	0.1977	-	ns	ns	9.27^{***}	-4.36^{*}

Allele: G – wild, A – mutation. Mean and SE were estimated using GLM model, values with the same superscripts belong to the same statistical group (A, B = p<0.01; a, b = p<0.05), P-value in GLM significant *p< 0.05, ** p<0.01, *** p<0.001, ns – not significant, X2P - covariate for weight of the right side of the carcass
